# Detection of experimental cartilage damage with acoustic emissions technique: An in vitro equine study

**DOI:** 10.1111/evj.13132

**Published:** 2019-06-06

**Authors:** B. R. Shakya, A. Tiulpin, S. Saarakkala, S. Turunen, J. Thevenot

**Affiliations:** ^1^ Research Unit of Medical Imaging, Physics and Technology Faculty of Medicine University of Oulu Oulu Finland; ^2^ Medical Research Center University of Oulu Oulu Finland; ^3^ Department of Diagnostic Radiology Oulu University Hospital Oulu Finland; ^4^ Research Unit of Cancer and Translational Medicine, Anatomy and Cell Biology University of Oulu Oulu Finland

**Keywords:** horse, osteoarthritis, fetlock, acoustic emission, flexion, extension

## Abstract

**Background:**

In horses, osteoarthritis (OA) mostly affects metacarpophalangeal and metatarsophalangeal (fetlock) joints. The current modalities used for diagnosis of equine limb disorders lack ability to detect early OA. Here, we propose a new alternative approach to assess experimental cartilage damage in fetlock joint using Acoustic Emissions (AE).

**Objectives:**

To evaluate the potential of AE technique in diagnosing OA and see how AE signals changes with increasing severity of OA.

**Study design:**

An in vitro experimental study.

**Methods:**

A total of 16 distal limbs (8 forelimbs and 8 hindlimbs) from six Finn horses were collected from an abattoir and fitted in a custom‐made frame allowing fetlock joint bending. Eight fetlock joints were opened, and cartilage surface was progressively damaged mechanically three times using sandpaper to mimic mild, moderate and severe OA. The remaining eight fetlock joints were opened and closed without any mechanical procedure, serving as controls. Before cartilage alteration, synovial fluid was aspirated, mixed with phosphate‐buffered saline solution, and then reinjected before suturing for constant joint lubrication. For each simulated condition of OA severity, a force was applied to the frame and then released to mimic joint flexion and extension. AE signals were acquired using air microphones.

**Results:**

A strong association was found between the joint condition and the power of AE signals analysed in 1.5–6 kHz range. The signal from both forelimb and hindlimb joints followed a similar pattern for increased cartilage damage. There were statistically significant differences between each joint condition progressively (generalised linear mixed model, P<0.001) in limbs with in vitro cartilage damage of varying severity while the control limbs did not show any changes.

**Main limitations:**

Small sample size using in vitro, mechanically induced cartilage damage.

**Conclusion:**

The AE technique presented here could differentiate the severity of fetlock joint cartilage damage. The consistent results for each simulated condition suggest there is potential for this method in the diagnosis of OA.

## Introduction

Osteoarthritis (OA) mostly affects the distal limb joints in horses, especially the fetlock joint as it has the highest range of motion among all joints, bears enormous tensile, compressive as well as rotational forces during high‐speed exercises and has a relatively small cartilage surface area 1, 2, 3. Moreover, its structural composition helps the horse to move at high speed and contributes to shock absorption for stabilising distal limbs 4. While treatments to cure chronic OA do not currently exist, diagnosis of mild OA is desirable so that preventive measures can be taken in time to slow down or even stop the progression of the disease.

Arthroscopy is used for evaluation of OA in fetlock joints. However, due to necessities of anaesthesia, specialised equipment, skills and limitation in full arthroscopic access to the joint structure, other techniques like radiography, computed tomography, scintigraphy and ultrasonography are used as alternative modalities. However, they too have their share of limitations such as the inability to show soft tissues, the requirement of anaesthesia, the high occurrence of false‐positive results and off‐incidence artefacts, respectively 5, 6, 7, 8. Magnetic resonance imaging has been proven to be the best direct approach for 3D visualisation of articular cartilage; nonetheless, its use is still limited in horses due to high expense and availability of appropriate magnets 9. Development of alternative methodologies may overcome the limitations with cost effectiveness, accessibility and portability. Acoustic emission (AE) is a widely used methodology in mechanical engineering to determine materials’ crack initiations, their propagation and bearing defects. Furthermore, it is also used to monitor wear during the process of friction and lubrication 10, 11, 12. In medical studies, AE‐based techniques have been used to assess ligament damage, bone fracture and joint friction monitoring 13, 14, 15. AE techniques have been suggested for monitoring bone cement crack growth causing loosening of hip implants 16. Specifically in joints, crepitus is known to be associated with the alteration of cartilage, symptomatic of conditions such as OA. Recent studies have demonstrated the potential of AE to quantify these changes during sit‐to‐stand movements 17, 18.

This study investigates the potential of AE technique in medical diagnosis for the detection of simulated equine OA which has never been reported in horses before despite promising preliminary results in human subjects. The aims of this study were 1) to measure an index of joint friction as a non‐invasive solution for detecting early OA, and 2) document how acoustic signals changed with increasing simulated conditions of OA severity. We hypothesised that healthy joints with optimal lubrication slide quietly against each other, producing low acoustic signals, whereas joints with cartilage damage move unevenly, thereby producing higher acoustic signals. Thus, we wanted to investigate whether acoustic signal analysis offers a potential scientific basis for new, convenient, non‐invasive tools to monitor the dynamic integrity of joints.

## Materials and methods

### Sample collection

A total of 16 distal limbs (8 forelimbs and 8 hindlimbs) from 6 Finn horses (age 9.64 ± 5.24 years, mean ± s.d.) were collected from an abattoir immediately after the death of the animals. Each limb was identified with the help of a registration certificate of Finn horses available at the abattoir. All the limbs were stored at −20°C after collection. The limbs were thawed 1 day before the experiment in a cold room at the temperature of 5°C. Trimming of hair on the limb was performed prior to the experiment so that it would not affect the signals during measurements.

### Experimental procedure

**Figure 1:**
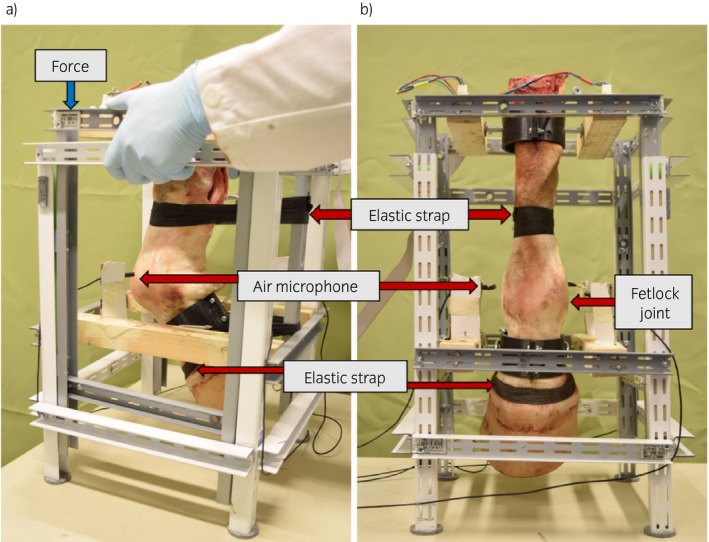
The distal limb of a horse fitted in a custom‐designed frame. a) Simulated flexion movement with applied force and b) simulated extension movement with the release of force.

**Figure 2:**
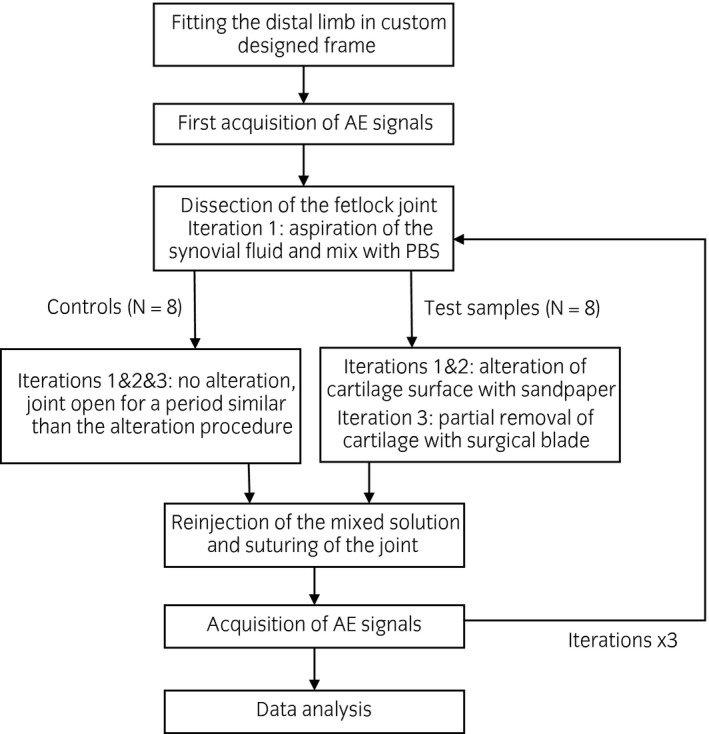
Block diagram showing the experimental procedure for osteoarthritis‐induced and control limbs.

A frame (Fig 1) was designed to hold the studied distal limb allowing flexion and extension motion of the fetlock to mimic normal joint bending. Each limb was fixed on the frame with screws placed at the proximal and distal ends of the cannon and the long pastern bones, respectively, allowing rotational motion of both bones at the fixation points. Two elastic straps were used, one above and one below the fetlock joint, to create a resisting force holding the limb in an extended position, simulating the force induced by the extensor muscle of the horse. A summary of the experimental protocol is shown in Figure 2. An external contact force perpendicular to the ground was applied manually from both sides in the upper part of the frame where the rails were attached (along with the main axis of the limb), which created tension in elastic straps to simulate the flexion motion of the fetlock joint. During application of the force, both cannon and pastern bones rotated at their fixation point, allowing an estimated 40° range of motion in the joint. Once the force was released, the limb returned to its original position automatically with the help of the elastic straps, which were acting as a resistance to withstand the applied force creating the extension motion of the joint. During such movements, acquisition of the acoustic signals was made to characterise different OA severities.

OA severities were simulated in 8 fetlock joints with a surgical procedure using the OARSI scoring system for guidance 19. The different stages assessed were based on cartilage damage: normal joint (grade 0/1 – intact surface), mild OA (grade 2/3 – surface discontinuity/vertical fissures, 1st iteration), moderate OA (grade 3/4 – vertical fissures/erosion, 2nd iteration) and finally, severe OA (grade 5 – denudation, 3rd iteration). Firstly, signals were acquired before the surgical procedure to monitor the response of a healthy joint (Fig 1). In the first iteration, the joint was dissected from the dorsal aspect and an incision was made on the synovial capsule to access the articular cartilage. The articular cartilage was visually checked to make sure it was intact, then the surface at the end of the cannon bone was rubbed once (one anterior‐posterior movement), by applying manual force with sandpaper (P60) at both lateral and medial condyles at dorsal region to simulate mild osteoarthritis in the joint (minimal cartilage damage visible). Two millilitres of synovial fluid was aspirated from the joint before wearing off the cartilage. The aspirated synovial fluid was mixed with the same amount of phosphate‐buffered saline (PBS). The PBS was added so that an adequate amount of fluid could be injected back for constant joint lubrication during the whole experimental procedure. After mixing, 2 mL of the solution was injected back into the joint after the wearing of cartilage was done. The joint was then closed with a 3/8 circled 30 mm eyeless suture of size 3‐0 using simple continuous pattern to maintain its integrity, and the acoustic signals were acquired. The same procedure was repeated to simulate moderate osteoarthritic condition during the second iteration. The cartilage surface was rubbed one more time with an increase of the applied force (depth of scratches higher than previous). One millilitre of solution was reinjected for compensating the fluid loss during the surgical procedure. In the third iteration, some portion of cartilage (N = 9 per joint, each 4 mm in diameter) were removed across the cartilage surface using a stainless surgical blade (size 23) to simulate severe OA. Similarly, as before, 1 mL of solution was reinjected to the cartilage surface to ensure constant joint lubrication. The same person was involved during the whole experiment to ensure a consistent level of cartilage wearing off. Fetlock joints from 8 additional limbs served as controls (Fig 2), which followed same experimental procedure without any alteration of cartilage surface to ensure there was no effect on acquired signals due to multiple opening and closing of the joint.

### Data collection

The acquisition of the joint acoustic signals was conducted with air microphones during the evening in a quiet room to reduce any disturbing noise that could affect the signals. Our system consisted of four air microphones (AT 899)^a^ with a frequency response of 20–20,000 Hz and having a dynamic range of 108 dB, placed crosswise in four corners of the custom‐designed frame at a distance of 3 cm away from the fetlock joint. Although four air microphones were used, the results of only one air microphone placed left on the front was used in this study. These air microphones were connected to power modules (AT 8537)^a^ which helps to convert the 11–52 VDC phantom power into a smaller DC bias voltage used to power the field effect transistor impedance matching circuit inside the microphone. The power modules were connected to an audio interface incorporating high‐quality analog pre‐amplifiers (Scarlett 18i8, 50 dB gain)^b^ which amplifies the acquired signals, as the acoustic signals generated by the motions of the joint are very low. A total of 10 signals from each simulated condition of the joint were recorded. Each signal consisted of two complete cycles of motions of the fetlock joint: one for flexion and the other for an extension. For distinguishing the start and end of each motion, a tri‐axial accelerometer (ADXL345)^c^ was used, placed at the top of the custom‐designed frame. The corresponding signals from the accelerometer were acquired simultaneously with the acoustic signals.

### Data analysis

**Figure 3:**
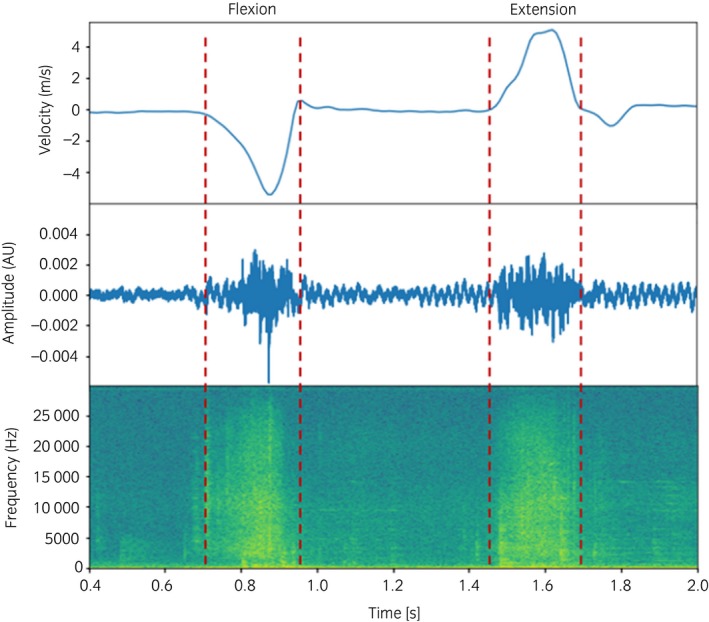
Example of frame velocity (top) used to segment the acoustic emission signal during flexion–extension movement (middle) and its corresponding representation in the frequency domain (bottom).

The information obtained from the accelerometer was used to calculate the velocity of the frame so that each motion of the joint can be automatically segmented (Fig 3). For analysing the segmented signals, a custom‐made algorithm was created using ad hoc developed Python software. The signals were acquired at the sampling rate of 192 kHz to average out noises for improving the signal‐to‐noise ratio and reducing aliasing for better phase response. After segmenting the AE signal for each motion, five per cent of its time at the start and end of the signal were deducted. The deduction was made to exclude any noise which may have been generated due to the collision of the upper frame with the lower base during motions of the joints. The acquired signals were then converted into the frequency domain using a fast Fourier transform (FFT) to observe the frequency distribution of the signals. Finally, the power of the segmented AE signals was calculated from FFT data using Parseval's relation for the discrete Fourier transform.

The Shapiro–Wilk test was used to check the normality of data, while box and whisker plots were used to monitor the distribution of data among each condition of the limbs. A generalised linear mixed model with repeated measures was used to evaluate the differences between the two groups (control and OA simulated) and between each iteration in both groups. Comparison between two groups was made based on each iteration of the joint conditions. This model was chosen so that the multiple limbs from the same horse can be reliably compared. In the model, iterations (i.e. normal joint, 1st, 2nd and 3rd iteration), groups (control or OA simulated) and limbs classified into two effects that is limb ends (forelimb or hindlimb) and limb sides (right limb or left limb) were set as fixed variables while horses were set as random variable. The power values for flexion and extension of each limb was set as dependent or target variable and repeated measurements for each iteration along with two effects of limbs (limb end and limb side) within horses were treated as repeated measures. The estimated means for different iterations were obtained from the fitted model, and the main effects between the iterations and groups were compared. Sequential Bonferroni test was performed to find out whether statistically significant differences exist between the control and OA‐induced limbs. Estimates of fixed effects were used to compare iterations for each group. Statistical significance was considered for P<0.05. Statistical analysis was performed using IBM SPSS statistics for windows, version 22^d^.

## Results

**Figure 4:**
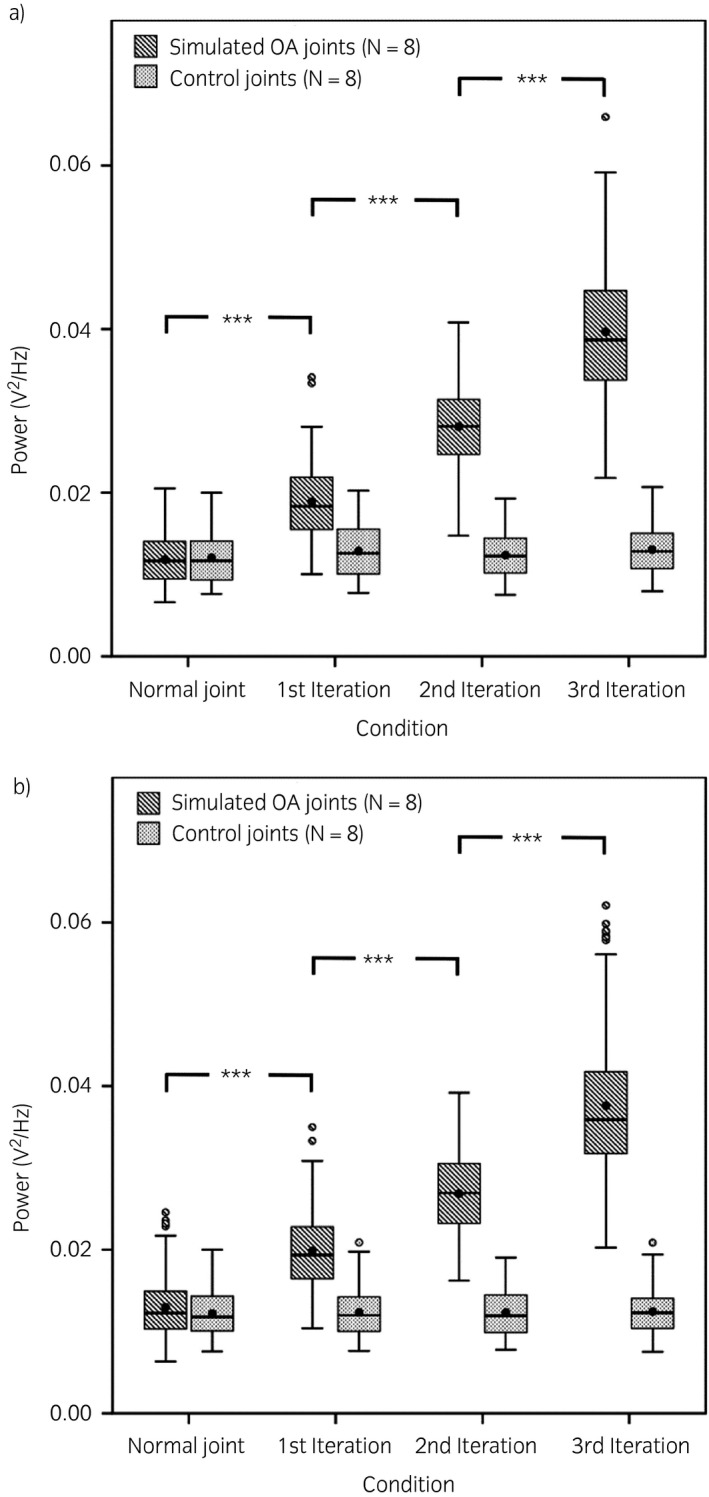
Distribution of acoustic emission power values for osteoarthritis (OA)‐induced and control limbs with each iteration of OA‐induced limbs corresponding to an increase in cartilage damage. a) flexion b) extension. For each plot, the box represents upper and lower quartiles, the line and black dot within the box represent median and mean respectively, the top and bottom whiskers represent the highest and lowest value excluding outliers, the white circles represent the outliers. ***P<0.001.

After visually observing the spectrums of different simulated OA severities, the spectral range 1.5–6 kHz was found to be most representative of the increase in power spectral density during each iteration of cartilage alteration, hence showing strong association with the joint condition. Among the data measured from each limb, some datasets were not normally distributed so generalised linear mixed model was used in the study as this model extends the linear model so that the target variable can have non‐normal distribution. The median value along with interquartile range (IQR) of control and OA‐induced limbs after each increased OA severity are listed in Table 1. Statistically significant differences (P<0.001) were observed between two groups after increased OA severity. Moreover, in the OA‐induced limbs, for each iteration of increased simulated OA severity, statistically significant differences (P<0.001) were observed between every joint condition. Within control limbs, there was no statistically significant difference between iterations (Fig 4).

**Table 1:** Median (IQR) power values of control and OA‐induced limbs for each iteration of joint condition during flexion and extension (unit: V^2^/Hz)

Iterations	Flexion			Extension		
	Control limbs	OA‐induced limbs	*P‐value	Control limbs	OA‐induced limbs	*P‐value
Normal joint	0.011 (0.004)	0.011 (0.004)	0.9	0.011 (0.004)	0.012 (0.004)	0.9
1st Iteration	0.012 (0.005)	0.018 (0.006)	<0.001	0.011 (0.004)	0.019 (0.006)	<0.001
2nd Iteration	0.012 (0.004)	0.028 (0.006)	<0.001	0.011 (0.004)	0.026 (0.007)	<0.001
3rd Iteration	0.012 (0.004)	0.038 (0.010)	<0.001	0.012 (0.003)	0.035 (0.010)	<0.001

* Differences between two groups (control and OA‐induced limbs) using sequential Bonferroni test.

**Figure 5:**
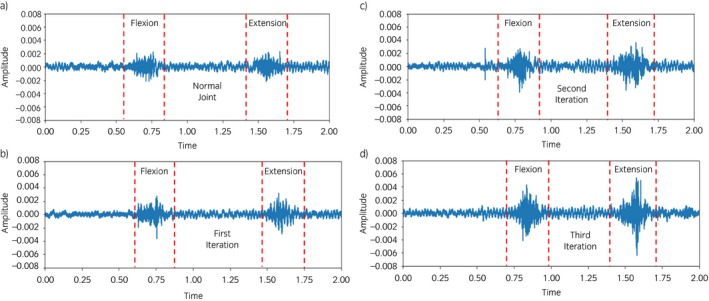
Waveform of the acoustic emission signals showing a change in amplitude with increasing cartilage damage. a) normal joint (control) b) the first iteration simulating mild osteoarthritis (OA) c) second iteration simulating moderate OA d) third iteration simulating severe OA.

Furthermore, the values for power obtained from each limb for both movements (flexion and extension) had high similarities among different conditions. In comparison with the healthy joint, the joint affected with simulated OA generated signals with higher amplitude, as shown in Figure 5.

## Discussion

This is the first study investigating the use of AE technique for detection of OA in distal horse limbs. Since no studies have yet been conducted to evaluate the effectiveness of this technique in controlled environment to diagnose OA, the aim of the current study was to provide a novel alternative method to detect OA, even at a mild stage of the condition. The AE technique has previously been reported for detecting OA in human knees 20, 21, 22, and OA knees produced consistently and significantly more AE events with higher amplitudes and longer duration than healthy knees 20. In Shark and co‐worker's study 21, the discrimination was done using the four‐phase model of sit‐stand‐sit movement. The results obtained from statistical parameters comprising of AE events, peak magnitude and average signal level showed that OA knees produced consistently and significantly more AE events with higher peak magnitude and average signal level values than healthy knees. In Sarillee and co‐worker's study 22, discrimination was done using wavelet packet transform. They obtained an accuracy of more than 80% after principal component analysis using a feed forward neural network and support vector machine. These studies used statistical parameters and digital signal processing in wavelet domain to differentiate the OA knees from healthy knees, while we also used digital signal processing but in the frequency domain based on frequency response of segmented AE signals.

In our study, the results obtained by calculating the power of AE signals from OA‐induced limbs for flexion and extension movements showed a clear difference between healthy and simulated osteoarthritic joints consistent with our first hypothesis. The power of acquired acoustic signal increased with simulated severity of OA, which fulfilled the second objective of our study to document how acoustic signal behaves with increasing severity of OA. The changes in chondral structure induced by sandpaper causes an increase of friction in the joint structure, leading to an increase in the power of the acoustic signal with each iteration of the cartilage alteration process. Figure 4 shows that every horse with mechanically induced cartilage damage followed a similar pattern for each condition during both movements, supporting both the repeatability of the protocol and our hypothesis.

Control limbs were used to demonstrate that the increase in friction obtained during the experiment was not the result of the drying of the joint or any other changes caused by the surgical procedure. We see no effect of multiple opening and closing of the fetlock joint on acquired AE signals (Fig 4). Repeated measurements for all distal limbs produced consistent power values and although there are overlaps between each iteration, a strong association between the power of the acoustic signal and the joint condition was observed.

The rise in amplitude of the AE is due to biomechanical disturbances from the wearing of the articular cartilage and its increased surface roughness, hence leading to higher friction in the area. The signal values between each simulated condition were significantly different, suggesting that changes in the joint structure due to deterioration of cartilage can be detected and quantified with the method introduced in this study.

Although we have data from only eight distal limbs, the method showed excellent consistency in the distribution of the data for each condition and differentiating mild OA from the healthy joints. Though the results reported here are promising, our study has some limitations. The number of distal limbs used here was limited. Since air microphones were used, ambient background noise and interface noise generated by the frame were also captured. While these noises affected the robustness of the method to some extents, the overall results of the experiment confirmed that they did not prevent the detection of cartilage damage, suggesting the potential for an in vivo application of the method. The extension movement was the most challenging to repeat, as the position of elastic straps and their elasticity was difficult to maintain across the different limbs. However, despite some slight variations in the obtained trends during extension, the results obtained were similar among legs. Furthermore, if veterinary applications are considered, the impact of the hooves striking the ground during a gait as well as other factors (e.g. brushing and forging) will have to be appropriately addressed using preprocessing, as they were not present in this study.

In conclusion, using AE technique, we were able to detect mechanically induced cartilage damage in an experimental setting and were able to document changes in AE signals with increasing severity of cartilage damage. This study is the first step towards developing AE as a non‐invasive and cost‐effective diagnostic tool for the detection of OA. However, in vivo applications will require the development of an equine friendly device with embedded and robust sensors and address challenges such as ambient noise and hooves strike artefacts. Additionally, in this study, only the cartilage surface was altered to simulate OA, but there are multiple in vivo changes associated with OA, such as a change in synovial fluid volume and the changes occurring in subchondral bone structures. It is possible that these processes might lead to more friction in joint and thus might give even more distinctive values than here. Therefore, our future studies will focus on developing and validating an in vivo acquisition system based on this method.

## Authors’ declaration of interests

No competing interests have been declared.

## Ethical animal research

Research ethics committee oversight not currently required by this journal: the study was performed on material obtained from an abattoir.

## Owner informed consent

Not applicable.

## Sources of funding

Business Finland (TEKES, grant no. 1241/31/2016) and Sigrid Juselius Foundation.

## Authorship

B. Shakya contributed to study design, study execution, data analysis and interpretation, and preparation of the manuscript. A. Tiulpin and J. Thevenot contributed to study design, data analysis and interpretation, and preparation of the manuscript. S. Saarakkala and S. Turunen contributed to study design and preparation of the manuscript. All authors gave their final approval of the manuscript.

## Manufacturers' addresses

^a^ Audio‐Technica US, Inc., Stow, Ohio, USA.

^b^ Focusrite Audio Engineering Ltd., High Wycombe, Buckinghamshire, UK

^c^ Analog Devices Inc., Norwood, Massachusetts, UK.

^d^ IBM, Armonk, New York, USA.
